# Pulmonary artery debanding in the cath lab: Lessons learned!

**DOI:** 10.3389/fcvm.2022.950123

**Published:** 2022-12-15

**Authors:** Rana Zareef, Sally Al Hassan, Nour Younis, Theresia Tannoury, Issam El Rassi, Fadi Bitar, Mariam Arabi

**Affiliations:** ^1^Faculty of Medicine, American University of Beirut Medical Center, Beirut, Lebanon; ^2^Brigham and Women’s Hospital and Harvard Medical School, Boston, MA, United States; ^3^Division of Pediatric Cardiology, Department of Pediatrics and Adolescent Medicine, American University of Beirut Medical Center, Beirut, Lebanon; ^4^Department of Surgery, American University of Beirut Medical Center, Beirut, Lebanon

**Keywords:** congenital heart disease—cardiac, pulmonary artery banding, pulmonary artery debanding, balloon debanding, interventional cardiac catheterization (ICC)

## Abstract

**Background:**

Although primary definitive repair of congenital heart disease has become the preferred management approach, pulmonary artery banding (PAB) remains a valuable palliative procedure used to restrict pulmonary blood flow in certain conditions. However, when the band is to be removed, another surgical intervention is usually required.

**Methods:**

To describe percutaneous removal of pulmonary artery band, the medical records of patients who underwent this procedure were reviewed.

**Results:**

Between 2000 and 2020, 143 patients underwent PAB. Of these, we attempted balloon debanding of the pulmonary artery in four patients. At the time of the procedure, the average age of patients was 36 ± 6.24 months, and their average weight was 12.37 kg. Band removal *via* catheter was successful in three cases and was associated with an adequate reduction in pressure gradient across the pulmonary artery band site (average of 71.67 ± 12.58 to 23.67 ± 2.89 mm Hg). None of the patients experienced complications during or after the procedure. Follow-up data after discharge (3–10 years) provides reassuring and satisfactory results.

**Conclusion:**

Based on our findings, we suggest that percutaneous removal of the pulmonary artery band might be a safe and effective alternative to surgical debanding. However, studies with a larger sample are required for further clinical implementation of the technique.

## 1 Introduction

The use of pulmonary artery banding (PAB) in the management of congenital heart disease (CHD) dates to the 1950s. The first PAB surgery was performed in 1952 on a 5 months old infant with a large ventricular septal defect (VSD) (1). The goal of banding was to limit excessive blood flow to the lungs in young infants. This would delay the corrective surgery until a desirable age or weight is achieved. Since its introduction, the use of PAB has allowed a significant increase in the survival rate of several congenital heart lesions that are associated with neonatal heart failure such as tricuspid atresia (2, 3). Although the use of PAB has been widely replaced by primary surgical repair of the cardiac anomaly, PAB continues to be a valuable technique employed in managing both simple and complex CHDs. It offers palliative treatment for children with left to right shunts and excessive pulmonary blood flow (4). Similarly, in complex CHD requiring a staged-approach, PAB serves as a bridge for definitive correction (4).

Indeed, around 2% of congenital heart lesions still require banding of the pulmonary artery (5). The advancement in prenatal detection techniques and management strategies of neonates admitted to the neonatal intensive care units have allowed an increased number of neonates with CHD requiring early surgical intervention. Ultimately, PAB has been increasingly useful in early management of such critically ill neonates (5, 6).

Nevertheless, the mortality and morbidity associated with this procedure, although improved over the years, remain significant (7). Besides, a noteworthy concern in PAB is that it becomes restrictive and limiting pulmonary blood flow as the patient grows and eventually requires dilation or complete removal (8). This usually entails another surgical intervention and cardiopulmonary bypass to remove or adjust the band, which carries a risk of mortality and morbidity.

To avoid surgical re-intervention, several research groups discussed the possibility of catheter-based band removal or dilation. Early studies were performed *in vitro* and on animal models and have yielded promising results. However, they were limited by the technical difficulties (9, 10). Clinical efforts to replace surgical debanding with balloon-based technique started as early as 1990. Nevertheless, the need for advanced equipment and materials has thwarted the implementation of this technique. As medicine advanced, with significant advancements in techniques and medical resources, some studies reported successful cases of catheter-based PA band removal or dilation (11–15).

To explore the significance of percutaneous pulmonary artery band removal, this article aims to describe and assess this technique along with its short- and long-term outcomes. It was performed at the Children’s Heart Center (CHC) at the American University of Beirut Medical Center (AUBMC), one of the leading tertiary referral centers in Lebanon and the Middle East.

## 2 Materials and methods

After securing Institutional Review Board (IRB) approval, we conducted a retrospective review of the medical records of patients who underwent PAB for various congenital heart conditions in the last 20 years at our institution. We explored those who had subsequent percutaneous removal of the band. The medical records of these patients including the pre-and post-procedure echocardiography reports, progress notes, surgical report, post-procedure notes, discharge summary, and catheterization reports were reviewed.

### 2.1 Procedures

#### 2.1.1 Banding technique

**FIGURE 1:**
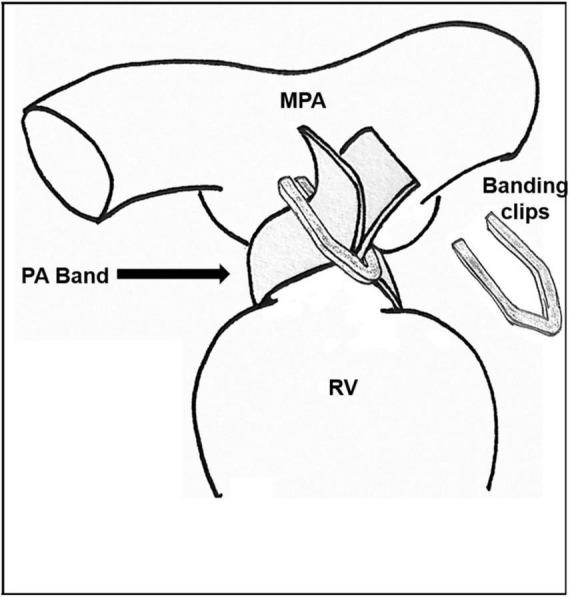
Pulmonary artery banding technique. RV, right ventricle; MPA, Main pulmonary artery; PA, Pulmonary artery.

The banding procedure, as described in Figure 1, may be performed through thoracotomy (anterior, lateral), or through a partial or full sternotomy. Any patent ductus arteriosus is ligated. The pulmonary artery is encircled with a 3 mm band, taking care to position it a few mm proximal to the right pulmonary artery, and distal the pulmonary valve. The band is tightened progressively with 1 or more sutures, and/or with metal clips, until the distal measured PAP is decreased to 50–30% of the systemic pressure, according to the cardiac lesion, as well as the patient’s age and weight at the time of the procedure. Alternatively, Trusler’s rule can be followed, adding the necessary fine tuning to reach the required PAP. The band is fixed to the adventicia of the main pulmonary artery to prevent its migration toward the pulmonary arteries. The band material is chosen (absorbable or durable) according to the cardiac lesion and planned treatment strategy.

#### 2.1.2 Surgical debanding

Surgical debanding, when needed, is achieved during the cardiac procedure performed to repair the initial heart defect for which the patient was banded. The band is removed usually at the end of the procedure. The main pulmonary artery can be progressively dilated with Heggar dilators until the appropriate size is reached. Alternatively, it can also be dilated using an appropriately sized balloon. Surgical options for the restoration of an adequate main pulmonary artery include the use of an autologous fresh or bovine pericardial patch to enlarge the narrowed area. Alternatively, the pulmonary artery may be transacted at its narrowest diameter; the narrowed and fibrotic area is then totally resected, and the main pulmonary artery reconstructed by end-to-end anastomosis, with or without the use of a patch. Any narrowing of the right or left pulmonary arteries is usually enlarged with a patch.

#### 2.1.3 Catheter-based debanding

**FIGURE 2:**
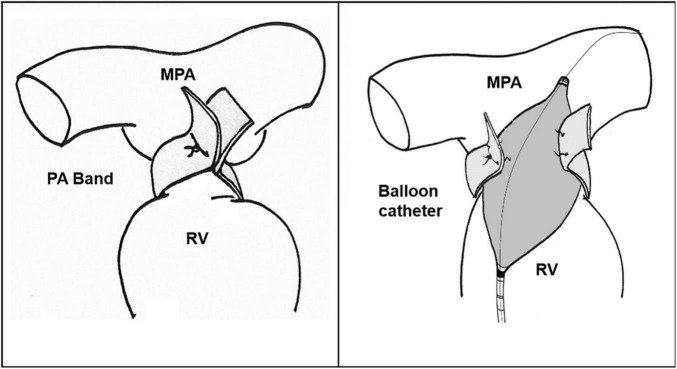
Pulmonary artery debanding *via* balloon catheter. RV, Right ventricle; MPA, Main pulmonary artery; PA, Pulmonary artery.

Catheterization procedures may be performed under sedation. Vascular access is secured percutaneously, and the sheath is advanced through the vein. Initially, hemodynamic evaluation is performed to measure the right heart, left heart and pulmonary circulation pressures. The guidewire reaches the main pulmonary artery proximal to the PA band. The size of the balloon is selected to be 1–1.2 times the size of the pulmonary valve annulus. An appropriately sized high-pressure balloon is advanced over the guidewire and is positioned across the PA band site. Under fluoroscopic guidance, the balloon is inflated until the inner clip is slipped, and the desired gradient is achieved (Figure 2). After dilation, the balloon is pulled back, the saturation and pressure are measured and the angiogram is repeated.

### 2.2 Statistical analysis

All analysis was conducted and represented using Microsoft Office Excel 2020, and GraphPad Prism 5.00.288. Discrete variables were represented as absolute numbers or percentages of the total. Standard deviations were calculated and included for all normally distributed continuous variables and were reported as mean ± standard error of the mean (SEM).

## 3 Results

During the period 2000–2020, a total of 143 patients underwent PAB. The mean age of the patients at the time of PAB was approximately 5.6 months. The male: female ratio was 1:0.8. 86 patients had subsequent band removal at our institution *via* thoracotomy. Multiple complications were encountered following the surgical removal of the pulmonary artery band: post-operative infection, hemothorax, pneumothorax, pericardial effusions, atrioventricular block, choreoathetosis, cardiopulmonary arrest requiring resuscitation with pressors and intubation, re-intubation, and ischemic strokes.

Percutaneous removal of the pulmonary artery band was attempted in four patients during the same period. Table 1 represents the demographic characteristics of these patients. Three patients had successful PA band removal *via* balloon catheter, while it was not possible in the fourth case, and the band had to be removed surgically. The mean age at the time of balloon angioplasty was 36 ± 6.24 months, the mean time between band placement and removal was approximately 32.6 months, and the average weight at the time of the procedure was 12.37 ± 3.7 Kg. The systolic gradient across the band site decreased from 71.67 ± 12.58 before band removal to 23.67 ± 2.89 mm Hg after the debanding. None of the three patients experienced post-procedure complications or required additional intervention. They didn’t require mechanical ventilation, or intensive care admission. After discharge, the patients were followed up at 2 weeks, at 1 month and then at every 3-month interval post-debanding. If the patients had reassuring follow-up results, they would be followed up annually thereafter. One patient was lost to follow-up after 3 years from the procedure, while the remaining two patients are still being followed up on, with the most recent visits 9 and 10 years after the band removal. Table 2 entails the procedural characteristics of the three patients. Below is a discussion of the four cases.

**TABLE 1:** Demographic characteristics of the patients.

Case	Sex	Age at banding (months)	Age at debanding (months)	Weight at debanding (Kg)	Underlying heart lesion
					
I	F	2	33	15	Large VSD
II	M	4	48	8.1	Multiple VSDs
III	F	4	27	14	Large VSD + hypoplasia of the transverse arch and descending aorta with a PDA supplying the descending aorta + coarctation of the aorta
IV	F	5	51	15.7	Large mid muscular VSD

F, female; M, Male; VSD, Ventricular septal defect; PDA, patent ductus arteriosus.

**TABLE 2:** Procedural details of the three patients who had undergone successful percutaneous PA band removal.

Case	I	II	III
Saturation before debanding (%)	100	98	100
Saturation after debanding (%)	100	100	100
RV systolic pressure pre-procedure (mmHg)	85	105	88
RV systolic pressure post-procedure (mmHg)	48	38	33
Pressure gradient across PA band pre-procedure (mmHg)	60	85	70
Pressure gradient across PA band site post-procedure (mmHg)	22	8	22
Vmax across main pulmonary artery pre-procedure (m/sec)	5.2	5.1	4.2
Vmax post-procedure (m/sec)	2.4	1.7	2.1
Follow up period	3 years	9 years	10 years
Most recent echo findings	VSD device in place, good LV and RV systolic function, RV systolic pressure of 40 mmHg, and mean gradient across the PA band site of 14 mmHg	Normal LV systolic function, multiple small apical muscular VSDs with left to right shunt, PA as well as its branches are of good size.	Vmax across MPA is 2 m/sec, RV systolic pressure of 35 mmHg, mean gradient of 8 across the PA band site. Pulmonary artery and branches are normal in size. Mild coarctation of the aorta with maximum velocity of 2.1 m/s

**FIGURE 3:**
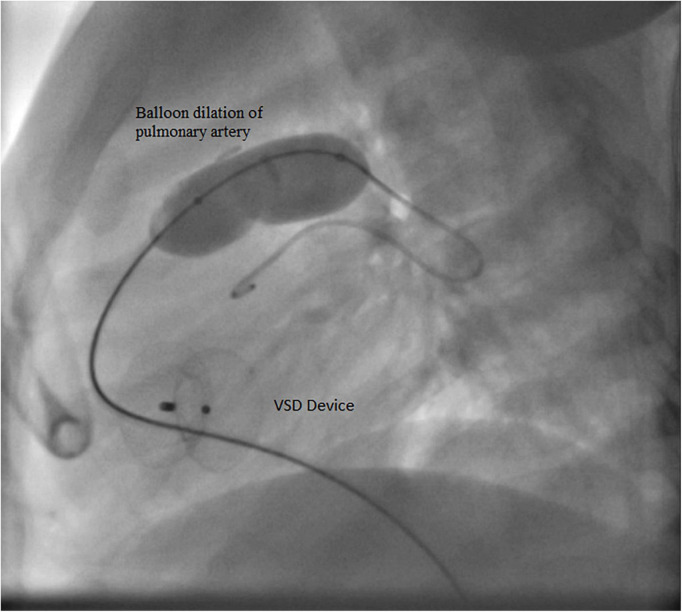
Balloon dilation of the pulmonary artery of Case I.

**FIGURE 4:**
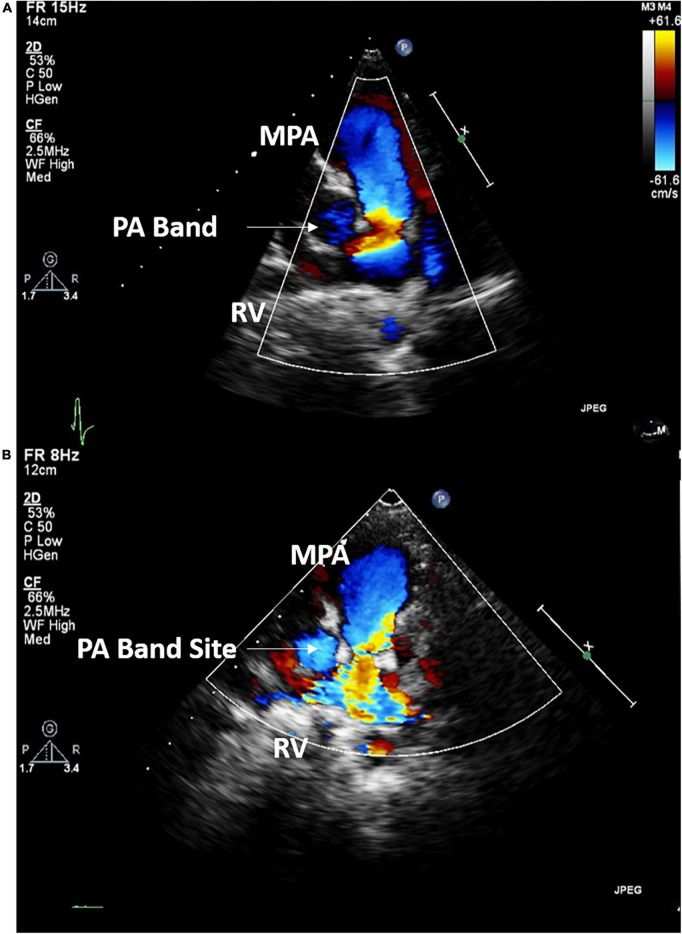
Echocardiographic imaging of Case I before **(A)** the debanding procedure and at 3-year follow up following the catheter-based debanding **(B)**. RV, right ventricle; MPA, Main pulmonary artery; PA, Pulmonary artery.

**Case I:** The first case of balloon dilation is a female patient who was diagnosed at birth with a large smooth muscular VSD measuring 13.5 mm in diameter and 7 mm in length. She had a PAB placed at the age of 2 months due to pulmonary overflow. At the age of 33 months, she presented for device VSD closure. Successful closure using a 16 mm amplatzer device was performed under general anesthesia without complications. Following the procedure, the right ventricular (RV) systolic pressure was measured at 85 mmHg, left ventricular (LV) systolic pressure was 95 mmHg, and the pressure gradient across the PA band was 60 mmHg. Three days later, balloon-based removal of the PA band was successfully performed under sedation, using 14 mm balloon (Figure 3). Pressure measurement following the procedure revealed: RV systolic pressure of 48 mmHg, and PA systolic pressure of 26 mmHg with a residual gradient of 22 mmHg across the band site. The patient tolerated the procedure well without complications and was pain-free by the second day following the operation, according to the Face, Legs, Activity, Cry, Consolability (FLACC) scale. She was followed up annually and when needed at the pediatric cardiology outpatient department *via* clinical and echocardiographic evaluation for 3 years following the percutaneous band removal; thereafter she was lost to follow up. At the last clinic visit, the patient was gaining weight adequately (weight = 20 Kg) and her systemic oxygen saturation was 100%. Her last echocardiographic imaging, at 3 years post-debanding, showed normal LV size and function, normal size of the main pulmonary artery, and an estimated RV systolic pressure of around 36 mm Hg. The patient was not maintained on any cardiac medications. Figure 4 shows the echocardiographic findings of the patient before the debanding procedure and at 3-year follow-up post debanding.

**FIGURE 5:**
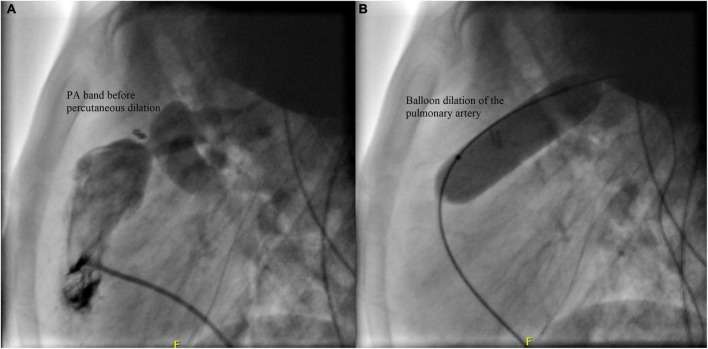
Angiographic findings of Case II before **(A)** and after **(B)** the debanding procedure.

**Case II:** A male patient was diagnosed at the age of 2 months with multiple apical muscular VSDs that are moderate to large. He had successful PA band placement at 4 months of age due to pulmonary overflow. He presented at the age of 4 years for catheter-based removal of his PA band. Before the procedure, echocardiography revealed severe narrowing of the PA with a systolic gradient of 85 mmHg across the band, an RV systolic pressure of 105 mmHg, and a PA systolic pressure of 20 mmHg. Following sedation, successful balloon dilation of the PA band was performed using a 15 mm balloon, which resulted in a reduced RV pressure of 38 mmHg, a residual gradient of 8 mmHg across the PA band, and a PA systolic pressure of 30 mmHg. On the same day post-debanding, the patient was free of pain according to the FLACC scale. No complications were encountered during or after the procedure, and he didn’t require further intervention. The angiographic findings of the patient before and after the angioplasty are shown in Figure 5.

**Case III:** A female patient was admitted to the AUBMC pediatric intensive care unit (PICU) at the age of 4 months with a picture of acute respiratory distress syndrome and was found to have a large apical muscular VSD measuring 16 mm with a left-to-right shunt, complicated by pulmonary overflow, a small patent foramen ovale, hypoplastic transverse arch, a PDA supplying the descending aorta, and coarctation of the aorta. She underwent coarctation repair and PA banding during the same admission. At 27 months of age, she was admitted for catheter-based PA debanding. Before the debanding, the PA pressure was found to be 64 mmHg. The procedure was performed under sedation. No complications occurred during or after the procedure, and she was discharged 1 day post-debanding.

The second and third cases are still followed-up annually at the pediatric cardiology outpatient clinic. At the last annual clinic check-up for case II, at 9 years post-debanding, the patient had a normal pulmonary arterial pressure of 35 mmHg. He was also gaining weight properly. Similarly, during the last follow-up visit for case III, 10 years after debanding, the patient was doing well with a good systolic function, a right ventricular systolic pressure of 35 mmHg, and a Vmax across the main pulmonary artery of 2.1 m/s. The systolic gradient across the PA band site was 17 mm Hg and the mean gradient was 10 mmHg.

Notably, these patients didn’t require intubation, were able to tolerate oral intake and mobilize within the same day of the procedure, and they only required paracetamol for pain control. The hospital stays for the three patients ranged from 3 to 7 days. The first case required 7 days of admission as balloon angioplasty was performed 3 days following the VSD device placement. Finally, the cost of balloon-dilation was around 8,000–10,000 U.S.D, which is around 17,000–20,000 U.S.D lower than the surgical removal.

**Case IV:** A female patient presented at 3 months of age with failure to thrive, recurrent infections, and fatigability upon feeding. She was found to have multiple apical VSDs and a large mid-VSD without any muscular or tissue covering. She later had a pulmonary artery band placed at 5 months of age due to pulmonary overflow. At the age of 5 years, she was admitted for diagnostic catheterization and possible balloon-debanding of the pulmonary artery, coupled with VSD closure. The patient underwent catheterization and debanding was attempted. The balloon was inflated without resolution of the band waist. The band could not be removed as multiple clips were used and placed in close proximity. The case was then converted to open heart surgery. The patient required intubation for 3 days and was discharged on day 5 post-debanding.

## 4 Discussion

A few studies have explored the use of pulmonary artery catheter-based debanding. All the studies were performed in developed countries. To our knowledge, this is the first study to report the use of this procedure at a tertiary center in a developing country like Lebanon.

To evaluate the efficacy and hospital course of the balloon-associated pulmonary artery debanding, our study retrospectively analyzed four cases who had attempted percutaneous pulmonary artery band removal. Three patients had successful percutaneous band removal, and all exhibited favorable outcomes. A decrease in the gradient across the PA band site was noted following the procedure in all patients. No complications during or after the catheter-based debanding were noted, and none required further interventions.

Variation in surgical practice, nevertheless, would impact the achievement of the procedure. The banding technique depicted in this manuscript is the usually implemented technique at our institution and is followed by many centers worldwide. Case IV had comparable demographic and baseline cardiac characteristics to the other patients who underwent catheter debanding; however, the debanding procedure couldn’t be done percutaneously as multiple clips were used to maintain the band. All three other successful cases had only one clip placed at the pulmonary artery band, which facilitated the percutaneous debanding. A tighter band maintained by a high number of clips may not be easily reversed with a balloon catheter. Therefore, upon band placement, the surgeon should consider the possibility of future percutaneous debanding.

Catheter associated debanding saves the patient from open-heart surgery and its associated morbidity and mortality, especially in patients with spontaneously closing lesions who are not scheduled for additional surgeries. The debanding procedure can also be coupled with VSD device closure to save the patient additional procedures. In case I, VSD closure and the debanding procedure were performed 3 days apart as this was the first percutaneous band removal at our institution; however, the two procedures can be performed simultaneously. Besides, in patients who require future surgical intervention, this approach can be used to delay the surgery. Furthermore, this newly adopted technique imparts smaller scars and reduces post-operative pain. In this case, paracetamol was the only medication used for pain control, compared to the need for multiple analgesics in the surgical debanding. In addition, none of the three patients required intensive care admission. The average hospital stay was 3 days, compared to the average stay of 9 days when the band is removed surgically at our institution. Likewise, our three patients were followed up for a relatively long period of time and the attained outcomes were encouraging. Two patients were followed up for 9 and 10 years, respectively. No significant stenosis was noted at the band site. They didn’t require any additional interventions.

In developing countries, management of CHDs through surgery is usually hindered by the shortage of medical equipment, specialized centers, personnel, and expertise. Indeed, a specialized CHD surgical team is not always available in low-income countries, and the schedule of congenital cardiac anomaly correction operations strongly depends on visiting teams, which negatively affects the prognosis (16). On the contrary, our approach relies on widely accessible balloon catheters; and can be easily employed in low-income countries.

The catheter-based procedure saved around 17,000–22,000 USD when it was compared to surgical removal of the band. In most developing countries, a proper national healthcare system is almost non-existent. Due to financial restrictions, treating common and communicable diseases, like infectious diseases, takes priority over congenital heart surgery. In Lebanon, for example, philanthropic organizations pay about 30% of the cost of CHD surgeries, with the rest usually covered by patients and the hospital (17). Therefore, replacing the surgical approach with percutaneous intervention solves a major financial challenge for these patients.

Theoretically, this procedure is not risk-free. Complications such as rupture of the pulmonary artery, dissection or aneurysm exist. In this study, we didn’t encounter any complications related to the procedure. Nevertheless, our data is extrapolated from a few patients. Hence, we argue that this technique should be studied in a larger population to assess its potential immediate and delayed complications.

The use of balloon catheters in band dilation or removal was described in previous studies as early as 1990. The first successful cases of pulmonary artery debanding *via* balloon catheter were described by Bjørnstad et al., in two patients (11). Brown et al., used dilatable bands to secure the ability to fully or partially expand the main pulmonary artery through balloon angioplasty in eight patients (13). No complications were noted during or after the procedure. They were able to record a significant decline in the gradient. Holmström et al. also performed successful catheter-based debanding on 17 patients (12). They suggested the use of a modified suture technique for the PA band that allows catheter-based debanding in the future. However, multiple complications were encountered during the procedure, including rupture of the pulmonary artery, temporary heart block requiring a pacemaker, contrast extravasation, and compromised coronary blood flow. *Morgan et al* also described two cases of balloon catheter debanding (18). A third case failed to dilate the balloon and was converted to open surgery; however, the patient had significant sub-valvular pulmonary stenosis. In another study, 12 successful percutaneous pulmonary artery debanding procedures were performed, two of which were for total debanding (19).

Many authors have discussed the use of dilatable bands or resorbable materials to avoid additional surgical intervention to remove the band. Unfortunately, such inventions didn’t gain wide clinical attention. To start with, some studies proposed the use of an adjustable pulmonary artery band, which would allow prompt adjustment of PAB according to the patient’s requirements. This would avoid surgical re-intervention for purposes of band adjustment. This approach relies on the use of a telemetric adjustable FloWatch-PAB pneumatic device to adjust the size of the PAB. By using this device, the authors reported a shorter intensive care stay and a smooth hospital stay. Nevertheless, the cost of the device remained a significant barrier (20, 21). Also, concerns have been raised regarding the need for additional surgical intervention when the removal of the device is necessary. Other possible reported complications such as pulmonary arterial aneurysm and dissection are also raised. All of this has restricted the clinical application of this technique.

Another suggested alternative practice relies on the use of absorbable PA band (22, 23). Bonnet et al., used polydioxanone bands to tighten the pulmonary artery (23). This technique spares patients the need for additional surgery for subsequent band removal. It is especially helpful in spontaneously closing lesions such as VSD. Unfortunately, the mean time for complete absorption of the band was 5.7–7.2 months following the operation, which may not be sufficient for complete resolution of the congenital lesions (22, 23). This limits the applicability of this technique in the dynamic cardiovascular system.

Finally, this study describes percutaneous pulmonary artery debanding along with the follow-up results. This procedure is feasible, inexpensive, and effective in securing the proper pressure goal. We propose that this technique might safely replace surgery-based band removal when performed by a well-trained pediatric cardiology team. It may also be coupled with other corrective procedures such as VSD closure. This approach requires readily accessible medical resources and thus can be performed in specialized centers in developing and developed countries. Further studies are needed to adequately evaluate the use of balloon catheters for pulmonary artery dilation.

This study has several limitations and concerns. The main limitation is inherent to the study design: retrospective chart review. The study is further constrained by the sparse patient population. Additionally, many hospitals and institutes might not apply the banding approach described in this study. Additionally, the usefulness and effectiveness of this strategy may be constrained by the need for several sutures or clips to tighten the band. Besides, the fact that it was used to remove rather than to dilate the bands may limit its usefulness in patients who require band readjustment. We attest that a higher-quality study involving a larger number of patients followed-up for a longer duration is required to evaluate adequately the safety and efficacy of this procedure and determine the need for technique modification.

## Data availability statement

The raw data supporting the conclusions of this article will be made available by the authors, without undue reservation.

## Ethics statement

The Institutional Review Board at the American University of Beirut approved the study on August, 2020 under the ID: BIO-2020-0325. Written informed consent from the participants or their legal guardian/next of kin was not required to participate in this study in accordance with the national legislation and the institutional requirements.

## Author contributions

MA developed the idea of the manuscript. RZ, SH, and NY collected and analyzed the data. RZ, SH, NY, and TT wrote the first draft of the manuscript. MA, IR, and FB did the final editing. All authors contributed to corrections and adjustment of subsequent iterations of the manuscript and approved and agreed with the content.
